# Differences in Cytotoxic, Genotoxic, and Inflammatory Response of Bronchial and Alveolar Human Lung Epithelial Cells to Pristine and COOH-Functionalized Multiwalled Carbon Nanotubes

**DOI:** 10.1155/2014/359506

**Published:** 2014-07-24

**Authors:** Cinzia Lucia Ursini, Delia Cavallo, Anna Maria Fresegna, Aureliano Ciervo, Raffaele Maiello, Giuliana Buresti, Stefano Casciardi, Stefano Bellucci, Sergio Iavicoli

**Affiliations:** ^1^Department of Occupational Medicine, INAIL (Italian Workers' Compensation Authority) Research Area, Via Fontana Candida 1, 00040 Monte Porzio Catone, Province of Rome, Italy; ^2^Department of Occupational Hygiene, INAIL (Italian Workers' Compensation Authority) Research Area, Via Fontana Candida 1, 00040 Monte Porzio Catone, Province of Rome, Italy; ^3^INFN, National Laboratory of Frascati, Via Enrico Fermi 40, 00044 Frascati, Province of Rome, Italy

## Abstract

Functionalized MWCNTs are used in many commercial and biomedical applications, but their potential health effects are not well defined. We investigated and compared cytotoxic, genotoxic/oxidative, and inflammatory effects of pristine and carboxyl MWCNTs exposing human respiratory (A549 and BEAS-2B) cells to 1–40 *μ*g/mL of CNTs for 24 h. Both MWCNTs induced low viability reduction (by WST1 assay) in A549 cells and only MWCNTs-COOH caused high viability reduction in BEAS-2B cells reaching 28.5% viability at 40 *μ*g/mL. Both CNTs induced membrane damage (by LDH assay) with higher effects in BEAS-2B cells at the highest concentrations reaching 20% cytotoxicity at 40 *μ*g/mL. DNA damage (by Fpg-comet assay) was induced by pristine MWCNTs in A549 cells and by both MWCNTs in BEAS-2B cells reaching for MWCNTs-COOH a tail moment of 22.2 at 40 *μ*g/mL versus 10.2 of unexposed cells. Increases of IL-6 and IL-8 release (by ELISA) were detected in A549 cells exposed to MWCNTs-COOH from 10 *μ*g/mL while IL-8 increased in BEAS-2B cells exposed to pristine MWCNTs at 20 and 40 *μ*g/mL. The results show higher cytogenotoxicity of MWCNTs-COOH in bronchial and of pristine MWCNTs in alveolar cells. Different inflammatory response was also found. The findings suggest the use of *in vitro* models with different end points and cells to study CNT toxicity.

## 1. Introduction

Carbon nanotubes (CNTs), characterized by cylindrical shape and composed of carbon atoms, possess specific properties that make them useful for various consumer, medical, and industrial applications [1]. Given their increasing applications also in composite materials and in new areas, which will lead to higher human exposure, it is very important to elucidate their potential adverse health effects. Several* in vitro* studies demonstrated cytotoxic and genotoxic potential [2–7] and inflammatory effects [8–15] of CNTs. Pulmonary inflammation and fibrosis were demonstrated also* in vivo* on mice exposed to MWCNTs by pharyngeal aspiration [16] or aerosol [17].

The toxicity and reactivity of CNTs are influenced by their physicochemical properties such as length and diameter, surface area, tendency to agglomerate, dispersibility in media, impurities, and presence of metal catalysts, due to the method of production [18–22].

To implement CNT applications, particularly in biomedicine, it is possible to improve their solubility and dispersion by chemical treatments, such as acid functionalization or other methods, that make them biocompatible and able to cross cell membrane and deliver attached cargos into the cells. However, chemical functionalization seems to affect the toxicity of CNTs. Some of available studies demonstrated a CNT toxicity decrease; other studies showed a toxicity increase. Several* in vitro* studies, performed prevalently on SWCNTs, showed lower cytotoxic effects of functionalized form compared with pristine, due to their improved dispersibility [23]. Other studies show that the functional group can significantly affect cellular toxicity; in particular, Gutiérrez-Praena et al. [24] found on human endothelial cells (HUVEC) that acid carboxylic functionalized SWCNTs were more toxic than pristine SWCNTs.

Among the available studies on functionalized MWCNT toxicity, Magrez et al. [25] showed on human lung-tumour cell line that MWCNT toxicity increases when carbonyl, carboxyl, and hydroxyl groups are present on their surface. Coccini et al. [26], in a study on human astrocytes and lung epithelial cells exposed to pristine and moderately and highly functionalized MWCNTs, found higher cytotoxic effects for highly functionalized (hf) NH_2_-MWCNTs. Patlolla et al. [27, 28] observed that functionalized MWCNTs had higher cytotoxic and genotoxic potential compared to nonfunctionalized form. Zhang et al. [29] showed in RAW 264.7 macrophages that both pristine and functionalized MWCNTs induced cell viability reduction and MWCNTs functionalized with carboxyl (COOH) group induced also serious inflammatory responses, as indicated by the production of inflammatory cytokines. Pulmonary toxicity and inflammatory response after exposure to pristine and COOH- or NH_2_-functionalized MWCNTs were also found* in vivo* on intratracheally instilled rats [30]. Otherwise the study of Fraczek-Szczypta et al. [31] demonstrated on murine macrophages RAW 264.7 that functionalization process of MWCNTs decreases their cytotoxicity in terms of viability.

Cytotoxic and genotoxic effects of commercial pristine and OH- functionalized MWCNTs on A549 cells were evaluated and compared in our recent studies [5, 6]. Such studies showed different mechanisms of cytotoxicity between pristine and OH-functionalized CNTs, with membrane damage for pristine MWCNTs and apoptosis for MWCNT-OH, while similar genotoxic effects were shown.

Taken together the available studies pointed out that particle surface modification and dispersion status in biological medium are key factors in determining cytotoxicity of CNTs.

So far, the results of available studies on biological effects of nanomaterial (NM) exposure, although showing genotoxic, oxidative, and inflammatory effects, that may be implied in a carcinogenesis process, remain still uncertain and contrasting since often the physicochemical properties of the studied NMs are not well specified making it difficult to evaluate and compare the results obtained in different studies.

In the present study, we evaluate and compare the toxicity of industrially produced pristine and -COOH functionalized MWCNTs, studying cytotoxic, genotoxic/oxidative effects and proinflammatory response on two human respiratory cell lines: lung alveolar epithelial cells (A549) and bronchial epithelial cells (BEAS-2B). The aim was to identify an* in vitro* experimental model that uses different and complementary end points and different target cells to elucidate CNT toxicity and to evaluate the role of functionalization on the induced effects. Moreover, we used common commercial MWCNTs and low concentrations because the potential human exposure in the different applications is prevalently to commercial forms of CNTs and it is rather low.

The lungs represent the main potential target organ during manufacture and processing of nanomaterials [32] involving a large number of workers. So we specifically choose for our study human alveolar (A549) and bronchial (BEAS-2B) epithelial cells representing the main pulmonary cells and the most used cell types in inhalation toxicity studies. In particular A549 cells maintain many morphological and biochemical characteristics of pneumocytes type II [33] and BEAS-2B cells are targets of inhalation and play an important role in the maintenance of mucosal integrity as mechanical barriers against various particulates [34].

We characterized tested MWCNTs analyzing the specific physicochemical properties by TEM and DLS. The potential cytotoxic and genotoxic/oxidative effects were studied using WST-1 assay to evaluate cell viability, LDH assay to assess membrane integrity, and Fpg-comet assay to detect direct and oxidative DNA damage. In addition we evaluated inflammatory effects of CNT exposure detecting cytokine release by ELISA.

Our study could be useful to clarify, in two different respiratory cell lines, the potential health effects of commercial CNTs, also at low concentrations, and understand if chemical functionalization of MWCNTs, made to improve the dispersion, increases their toxicity before extending their applications particularly in the biomedical field.

## 2. Materials and Methods

### 2.1. Nanomaterials

Commercial pristine MWCNTs and functionalized MWCNTs-COOH purchased by HeJi (China) were employed. As specified by supplier, both CNTs were synthesized by chemical vapor deposition (CVD) which was followed by traditional acid treatment in the case of MWCNTs-COOH. The purity of pristine MWCNTs was up to 97.37% and impurities in the sample were: Cl 0.20%, Fe 0.55%, Ni 1.86%, and S 0.02%. The purity of MWCNTs-COOH was up to 97.46% and impurities present in the sample were Al 0.19%, Cl 1.02%, Co 1.09%, and S 0.04% (specifications given by the supplier). The functionalized nanotubes had -COOH > 5 wt%.

### 2.2. MWCNT Characterization

MWCNTs were analyzed using energy filtered transmission electron microscopy (EFTEM) to measure MWCNT diameter and length. MWCNTs were dispersed in water (1 mg/mL) and the suspension was diluted at 0.05 mg/mL and sonicated in two 5 min steps with a 30 s pause; then a drop of the nanotubes suspension was deposited on 300-mesh copper grids coated with a carbon film, to measure the nanotube lengths and the agglomerate diameters, and on 1000-mesh gold grids, to measure the nanotube diameters and to analyze MWCNT morphology and structure.

Conventional and high resolution TEM (HRTEM) micrographs were acquired and chemical elemental analysis was carried out by means of energy dispersive X-ray spectroscopy (EDS).

TEM experiments were performed by FEI TECNAI 12 G2 Twin operated at an accelerating voltage of 120 kV, equipped with an electron energy filter (Gatan Image Filter, BioFilter model), and a Peltier cooled charge-coupled device based slow scan camera (Gatan MultiScan camera, model 794IF). MWCNTs were suspended in cell media (0.1 mg/mL in RPMI or BEGM) and sonicated in two 5 min steps with a 30 s pause to measure the agglomerate/aggregate sizes by means of both TEM and dynamic light scattering (DLS). DLS analysis was performed using a Zetasizer Nano ZS (Malvern, UK). Using the same instrument, the Z potential of MWCNT suspensions (0.1 mg/mL in RPMI or BEGM medium) was detected.

Brunauer-Emmett-Teller (BET) specific surface area (SSA) analysis of tested MWCNTs was performed by Monosorb rapid surface area analyzer (Quantachrome Instruments, USA).

### 2.3. Cell Culture and Exposure

Human lung epithelial (A549) and bronchial epithelial (BEAS-2B) cell lines were obtained from the American Type Culture Collection (ATCC) (Rockville, MD). A549 cells were cultured in Rosewell Park Memorial Institute 1640 medium (RPMI 1640) (EuroClone, United Kingdom) supplemented with 10% fetal bovine serum (FBS) and BEAS-2B cells were cultured in Bronchial Epithelial cell Growth Medium (BEGM) BulletKit (Cambrex Bio Science Walkersville Inc.) both at 37°C in 5% CO_2_. Cells (8 × 10^4^ cells/well) were seeded into 24-multiwell culture plate (15.6 mm well diameter) and cultured for 24 h before the exposure.

Semiconfluent cell cultures were exposed for 24 h to pristine and functionalized MWCNTs. Cells were kept at 37°C in 5% CO_2_ during exposure time.

A stock solution (2 mg/mL) of each carbon nanotube, MWCNTs and MWCNTs-COOH, was prepared suspending nanotubes in distilled water. At moment of exposure the solution was vortexed 1 min and then sonicated 5 min to disperse the structures. From this stock, a working solution (1 mg/mL) of nanotubes was prepared in the complete culture media (RPMI 1640 with 10% FBS and BEGM) compatible with the* in vitro* systems used. The working solution was sonicated in two steps of 5 min with a pause of 30 secs, before being rapidly added to the cells with final concentrations of 1, 5, 10, 20, and 40 *μ*g/mL corresponding to 1, 5, 10, 20, and 40 *μ*g/cm^2^. Cells were kept at 37°C in 5% CO_2_ during exposure times. At least three different independent experiments were performed for each series of exposures. At the end of the incubation period, all cultures were examined and photographed before and after washing the cells with PBS buffer in an optical phase contrast Olympus IX50 microscope combined with a digital camera CANON EOS 500 D.

### 2.4. Quantitative Measurement of Cell-Associated MWCNTs

Quantitative measurement of MWCNT uptake was performed as previously described by Hirano et al. [11]. In brief, cells were cultured in 24-well plates and exposed to 40 *μ*g/mL of two tested MWCNTs for 2, 4, 8, 16, and 24 h; after exposure the culture medium was removed and the cells were washed two times with PBS and lysed with 0.4 mL of 0.2 M NaOH solution at room temperature for 2 h. Dimethyl sulfoxide (0.2 mL) was added to lysate and the lysate was pipetted up and down until MWCNTs were well dispersed. A 200 *μ*L sample was transferred to a 96-well plate and Optical Density (O.D.) at 642 nm was measured using a microtiter plate reader (Wallac Victor 2, Perkin Elmer, USA). Standard MWCNT samples (40 *μ*g/mL of tested CNTs in each culture medium) were prepared similarly without the cells and the relative O.D. was considered the total dose of exposure. Each experimental point was done in triplicate using duplicate wells per concentration and the data are represented as the mean ± SD. The ratio O.D. of exposed cells in respect to O.D. of total standard MWCNT samples was calculated and expressed as percent of dose.

### 2.5. Cell Viability (WST-1 Assay)

Cell viability of A549 and BEAS-2B cells exposed for 24 h to both MWCNTs was evaluated using the WST-1 (2-(4-iodophenyl)-3-(4-nitrophenyl)-5-(2,4-disulfophenyl)-2H-tetrazolium) assay kit (Takara Bio Inc., Shiga, Japan). We used this assay, assumed to be more reliable than MTT, to evaluate NM cytotoxicity. The water soluble tetrazolium salt WST-1, in contrast to MTT, is released into the supernatants without cell lysis and detected at 450 nm instead of 540 nm. The absence of cell lysis avoids the release of the CNTs present into the cells that could cause false cytotoxicity interacting with colorimetric indicator dye. Briefly, after exposure, the culture medium was removed and the cells were washed three times with PBS to avoid any interference in light absorption due to the CNTs. Then 500 *μ*L of fresh culture medium and 50 *μ*L of PreMix WST-1 solution were added to each cell culture well and incubated for 3 h at 37°C, protecting the plate from the light. At the end of incubation, 200 *μ*L of final mixture was transferred in an optically clear 96-well flat bottom microtiter plate. Formazan dye, formed by metabolically active cells, was quantified by measuring its absorbance (450 nm) using a microtiter plate reader (Wallac Victor 2, Perkin Elmer, USA). Unexposed cells were used as control.

Background and control were obtained by absorbance [*A*] measurement of culture medium and cell medium of unexposed cells, respectively. Data from CNT exposed cells are expressed as % of viable cells calculated by [*A*] tested sample/[*A*] control × 100. Each experimental point was done in triplicate using duplicate wells per concentration and the data are represented as the mean ± SD.

### 2.6. Membrane Integrity

Lactate dehydrogenase activity release, used as indicator of cell membrane damage, was measured by LDH assay kit (Cytotoxicity Detection Kit, Roche Diagnostics, Milan, Italy) on the culture medium of cells exposed to both MWCNTs for 24 h. Unexposed cells were used as control and cells exposed to 1% Triton X-100 were used as positive control of cytotoxicity. Following kit instructions, aliquots (100 *μ*L) of supernatant and reaction mixture containing catalyst and dye solution (iodotetrazolium chloride and sodium lactate) were transferred into corresponding wells of an optically clear 96-well flat bottom microtiter plate and incubated for up to 30 min at 15–25°C, protecting the plate from the light. The absorbance [*A*] was measured at 490 nm using a spectrophotometric microtiter plate reader (Wallac Victor 2, Perkin Elmer, USA). Background and control were obtained by LDH measurement of assay medium and unexposed cell medium, respectively. Total cellular LDH release was measured in cell lysates obtained by treatment with 1% Triton X-100 solution and represents the positive control. Data from unexposed (control) and exposed cells were calculated and expressed as percentage of cytotoxicity representing the mean of three independent experiments, each using triplicate wells per concentration. In particular for each experiment the cytotoxicity % was calculated using the following equation:

(1) $$\begin{array}{c} \text{Cytotoxicity}\;\%=\frac{\text{mean}\left[A\right]\text{value}\;\text{of}\;\text{exposed}\;\text{cells}-\text{mean}\left[A\right]\text{value}\;\text{of}\;\text{control}\;\text{cells}}{\text{mean}\left[A\right]\text{value}\;\text{of}\;\text{positive}\;\text{control}\;\text{cells}-\text{mean}\left[A\right]\;\text{value}\;\text{of}\;\text{control}\;\text{cells}}\times 100. \end{array}$$

### 2.7. Comet Assay

After exposure for 24 h the cells were washed with PBS then detached by trypsinization (0.25% porcine trypsin in 2% EDTA (Sigma-Aldrich, USA)), centrifuged, resuspended in 100 *μ*L of PBS, and analysed by Fpg modified comet assay immediately to evaluate simultaneously direct and oxidative DNA damage. According to the protocol, for each experimental point, two samples are prepared: one to be treated with Fpg enzyme (that recognizes and cuts the oxidized DNA bases) allowing to evaluate oxidative DNA damage and the other untreated sample allowing the detection of direct DNA damage [35]. Unexposed cells were used as control and cells exposed for 30 min to 100 *μ*M H_2_O_2_ were used as positive control. At least three independent experiments were performed for each exposure time at all the concentrations. The previously described protocol [6] was used. Images of 100 randomly selected comets either from Fpg enzyme treated or untreated slides, stained with ethidium bromide, were acquired and analyzed from each sample, with specific image analyzer software (Delta Sistemi, Rome, Italy).

Measurements of comet assay parameters such as % DNA in the tail, tail length, and tail moment (TM), representing the product of relative tail fluorescence intensity and tail length, were obtained from the analysis. For each experimental point we calculated the mean TM (tail moment from enzyme untreated cells), which indicates the direct DNA damage, and the mean TMenz (tail moment from Fpg-enzyme treated cells), which evaluates direct and oxidative DNA damage. Oxidative DNA damage was evaluated in terms of oxidized DNA bases (sites recognized and cut by Fpg) and calculated subtracting TM (direct DNA damage) from the TMenz (both direct and oxidative DNA damage), both in exposed and unexposed cells. The results were expressed as means of three independent experiments.

### 2.8. Detection of Cytokines

A549 and BEAS-2B cells were treated with MWCNTs and MWCNTs-COOH for 24 h. The cell supernatants were collected, centrifuged to remove any remaining CNTs, and then stored at −80°C. Cytokines were detected by using eBioscience assay kits (Vienna, Austria). Concentrations of the interleukine 6 (IL-6), interleukine-8 (IL-8), and tumor necrosis factor (TNF*α*) were determined by human enzyme-linked immunosorbent assay (ELISA) according to the manufacturer's guidelines. Cells incubated without nanomaterials were used as control. The absorbance was measured to 450 nm and quantified with a microplate reader (Wallac Victor2, Perkin Elmer, USA). In A549 cells, cytokine release was detected after 24 h exposure to 20 and 40 *μ*g/mL of pristine MWCNTs and to 1, 5, 10, 20, and 40 *μ*g/mL of MWCNTs-COOH. In BEAS-2B cells, cytokine release was analysed after 24 h exposure to 5, 20, and 40 *μ*g/mL of pristine MWCNTs and to 20 and 40 *μ*g/mL of MWCNTs-COOH.

### 2.9. Statistical Methods

Nonparametric Kruskal-Wallis test followed by post hoc T3 Dunnett's tests was used to assess the presence of statistically significant differences between exposed and control cells for cell viability (WST1 assay), membrane damage (LDH assay), and cytokine release (ELISA). Direct and oxidative DNA damage were assessed comparing mean TM values and mean TM differences (TMenz-TM) values, respectively, of exposed cells compared to unexposed cells using a nonparametric Kruskal-Wallis test followed by post hoc T3 Dunnett's and Bonferroni tests, and the significance of the difference was established for each experimental point.

To compare direct DNA damage induced by pristine MWCNTs in respect to that induced by functionalized MWCNTs, we calculated for each experimental point the increments of TM values of exposed cells in respect to unexposed cells (subtracting TM values of control cells from TM values of exposed cells). Then we used the nonparametric Mann-Whitney test to compare such TM increments. In addition, we used Mann-Whitney test also to compare the effects induced by each kind of CNT in A549 cells in respect to BEAS-2B cells. Student's* t*-test was used to compare Z potential, agglomerate size, and uptake of pristine and COOH-functionalized MWCNTs.

## 3. Results

### 3.1. Nanomaterial Characterization

**Figure 1:**
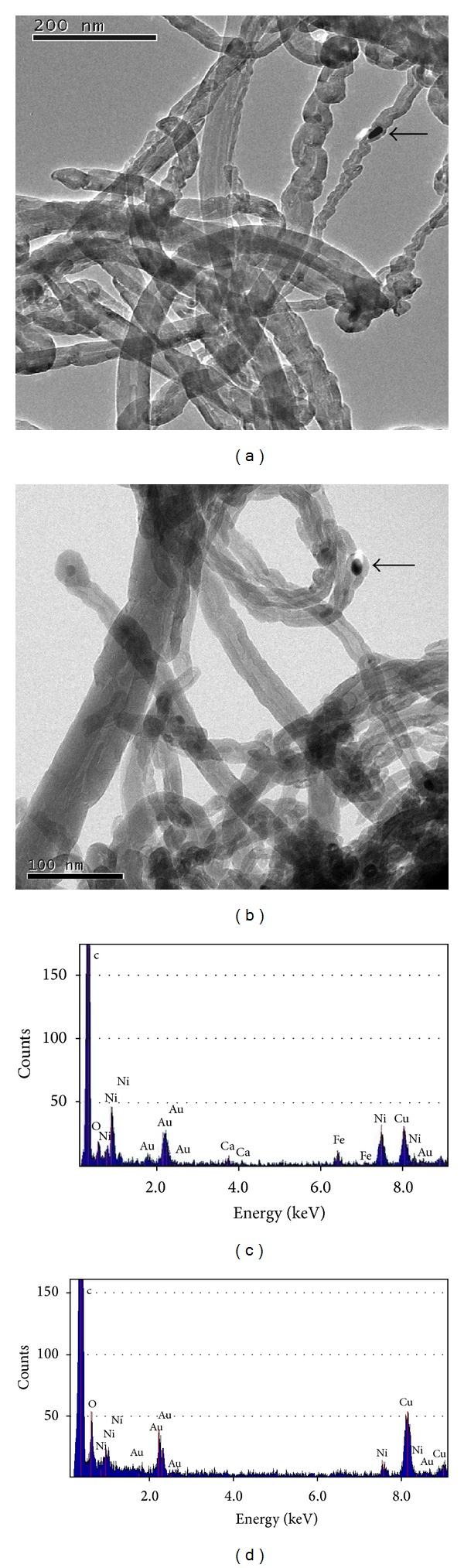
TEM images of pristine MWCNT (a) and MWCNT-COOH (b) deposited on a 1000-mesh gold grid to measure outside diameter. The images also show nanoparticle inclusion of the metallic catalyst nickel (indicated by arrow) used to synthesize the nanotubes by CVD (bar: 200 nm and 100 nm, resp.). EDS spectra of pristine MWCNT (c) and MWCNT-COOH (d).

The characterization of nanotubes structural parameters by TEM analysis revealed that pristine and MWCNTs-COOH are “bamboo-like,” without a defined inner channel. Moreover their outside diameter is not well defined and it changes abruptly along the nanotube itself (Figures 1(a) and 1(b)). The elemental analysis (Figures 1(c) and 1(d)) confirms the presence of nickel, used as metal catalyst in CNT synthesis by CVD, and shows higher amount of oxygen and lack of iron in MWCNTs-COOH (Figure 1(d)) as consequence of acid treatment used to functionalize CNTs. In the spectrum the gold peaks are due to the grid.

Table 1 shows the results of tested MWCNT characterization. The minimum value of the tube length has an indicative meaning, because nanotubes tend to break along their length, forming carbonaceous agglomerates of nanoparticles. The table shows smaller outside diameter (24.5 versus 32.0 nm), shorter length (0.029–1.56 versus 0.070–7.80 *μ*m), and larger SSA (139.1 versus 106.7 m^2^/g) of MWCNTs-COOH in respect to pristine MWCNTs.

The DLS findings also show that functionalized MWCNTs have a significantly more negative Z potential and smaller agglomerate sizes compared to pristine MWCNTs in RPMI medium with 10% FBS while in BEGM agglomerate sizes and Z potential of functionalized and pristine MWCNTs are very similar.

### 3.2. Nanotubes Dispersion after Exposure

**Figure 2:**
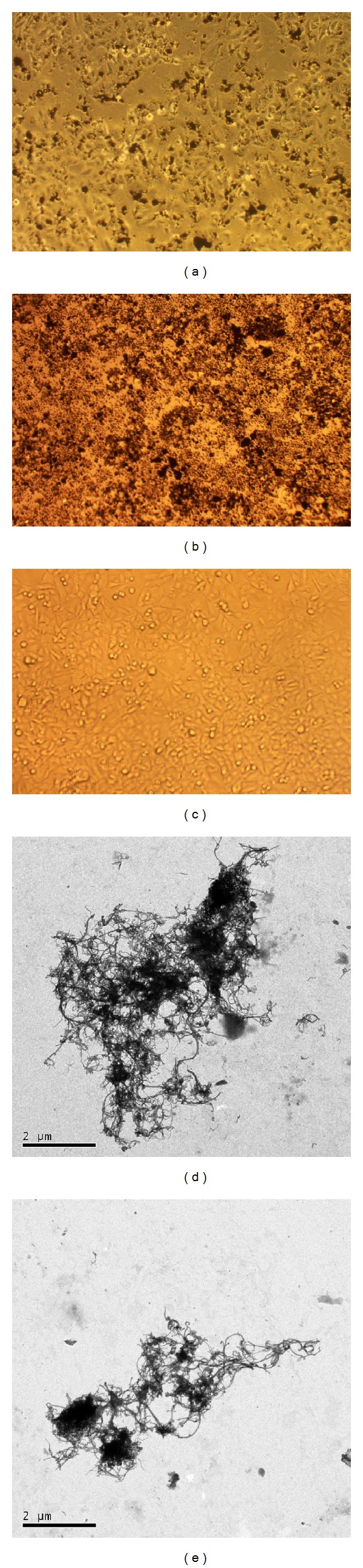
Representative optical microscopy images of A549 cells exposed for 24 h to CNTs before washing them to remove CNTs dissolved in the medium. (a) Cells exposed to 40 *μ*g/mL of pristine MWCNTs; (b) cells exposed to 40 *μ*g/mL of MWCNT-COOH; (c) control cells. Magnification: 20x. TEM micrographs of pristine MWCNTs (d) and MWCNT-COOH (e) agglomerates/aggregates in RPMI with 10% FBS culture medium (bar 2 *μ*m).

**Figure 3:**
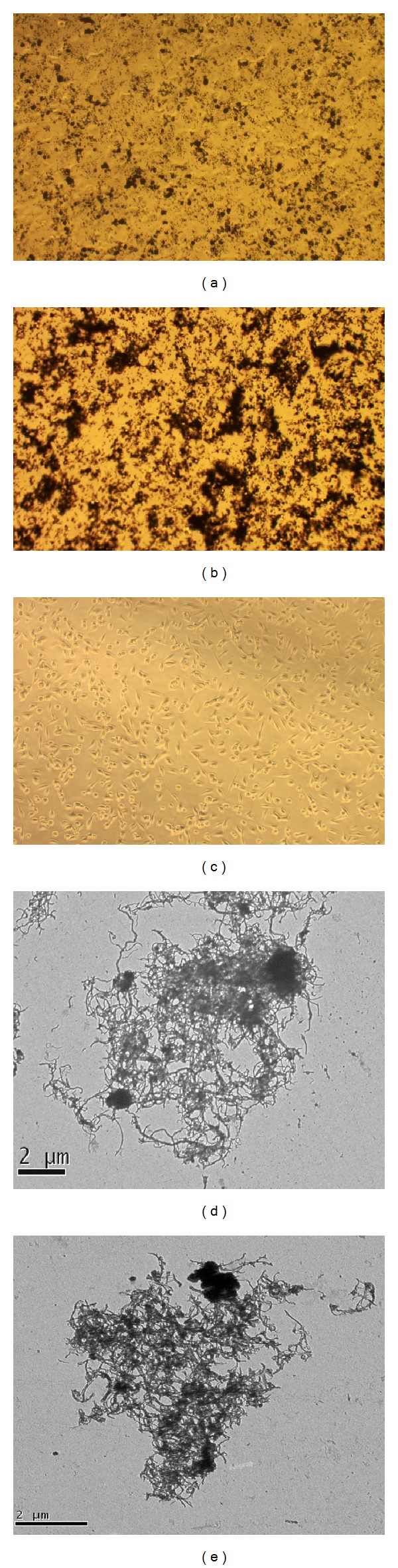
Representative optical microscopy images of BEAS-2B cells exposed for 24 h to CNTs before washing them to remove CNTs dissolved in the medium. (a) Cells exposed to 40 *μ*g/mL of pristine MWCNTs; (b) cells exposed to 40 *μ*g/mL of MWCNT-COOH; (c) control cells. Magnification: 20x. TEM micrographs of pristine MWCNTs (d) and MWCNT-COOH (e) agglomerates/aggregates in BEGM culture medium (bar: 2 *μ*m).

Optical phase contrast microscopy analysis showed that CNTs distribute differently after the exposure. Figures 2 and 3 represent examples of both cell kinds exposed to 40 *μ*g/mL of the tested MWCNTs. In particular, the pristine MWCNTs adhere to each other forming agglomerates on both cell types (Figures 2(a) and 3(a)). MWCNTs-COOH, as expected and in accordance with physicochemical characteristics, were better dispersed in A549 cell culture (performed in RPMI medium with serum) (Figures 2(b) and 2(e)) than in BEAS-2B cell culture (performed in BEGM medium) (Figures 3(b) and 3(e)). In fact, as showed in Figure 2(b), MWCNTs-COOH dispersed in RPMI generate a greater amount of small agglomerate/aggregates uniformly distributed in each culture well, compared with the most heterogeneous agglomerate/aggregates of MWCNTs-COOH dispersed in BEGM medium (Figure 3(b)).

### 3.3. Quantitative Measurement of Cell-Associated MWCNTs

**Figure 4:**
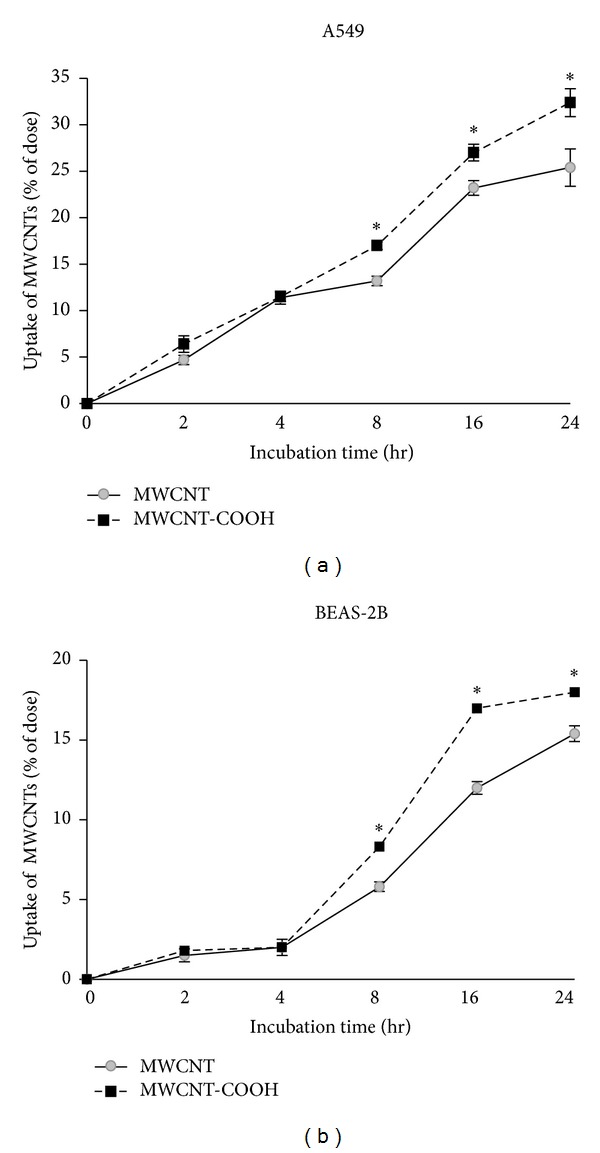
Percent association of pristine and COOH-functionalized MWCNTs with A549 cells in RPMI cell medium with 10% FBS (a) and with BEAS-2B cells in BEGM cell medium (b). The value of cellular uptake was expressed as percentage of total MWCNT added to the well (mean ± SD). ∗*P* ≤ 0.05, MWCNT-COOH versus MWCNTs.

The amount of MWCNTs reaching the cells (including MWCNTs adhering to the cell membrane and present inside the cells) was measured by O.D. value of exposed cells in respect to O.D. of total standard MWCNT sample and was expressed as percent of dose. The time-course of changes in MWCNT uptake by both cell types exposed to 40 *μ*g/mL of both MWCNTs is shown in Figure 4. In A549 cells (Figure 4(a)) the uptake of both MWCNTs was rather slow for the first 2 h and reached 11% within 4 h, up to 25% and 32%, respectively, for pristine and functionalized MWCNTs after 24 h. In BEAS-2B cells (Figure 4(b)) the uptake of both MWCNTs was very slow for the first 4 h and reached 15% and 18%, respectively, for pristine and functionalized MWCNTs after 24 h. It is important to note the rapid increase of uptake starting from 8 h with a significantly higher uptake for COOH-functionalized in respect to pristine MWCNTs and higher uptake of both MWCNTs in A549 cells.

### 3.4. Viability

**Figure 5:**
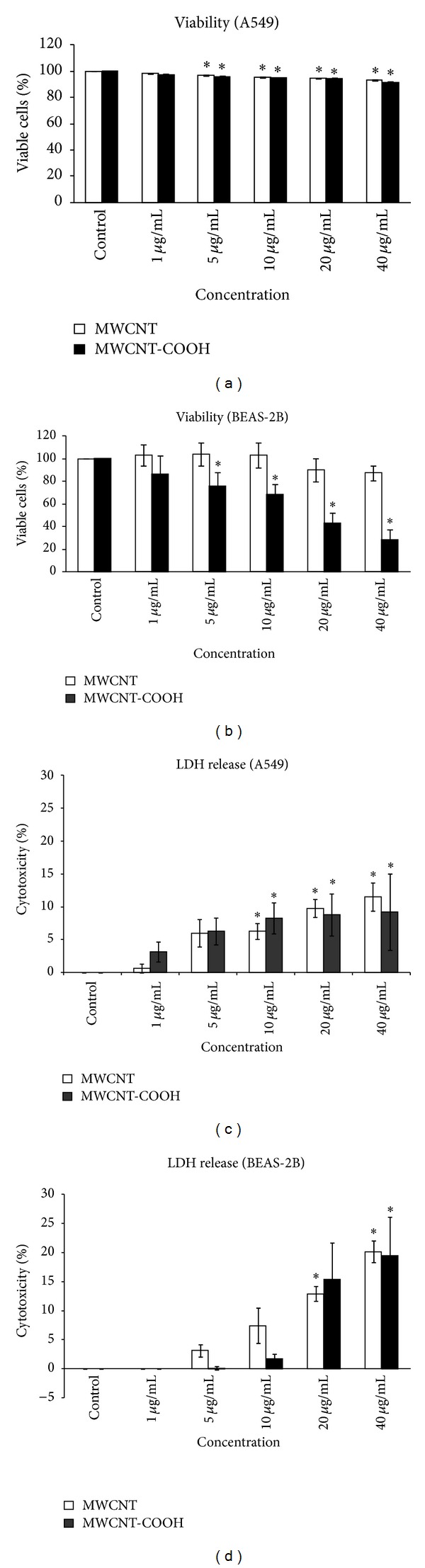
Cytotoxicity of A549 ((a), (c)) and BEAS-2B ((b), (d)) cells after 24 h exposure to pristine MWCNTs and MWCNT-COOH. ((a), (b)) Viability percentage; ((c), (d)) cell membrane damage. Data represent the means of three independent experiments. Unexposed cells were used as control. ∗*P* ≤ 0.05.

In A549 cells pristine and functionalized MWCNTs induced similar slight decrease of viability percentage in exposed as compared to the unexposed cells (Figure 5(a)). On the contrary, on BEAS-2B cells tested CNTs elicited different effects: MWCNTs-COOH induced a statistically significant dose-dependent reduction of viable cells beginning from 5 *μ*g/mL; pristine CNTs induced slight, not significant, decrease of viable cells only at the highest concentrations (Figure 5(b)). Mann-Whitney test, used to compare viability results in A549 in respect to BEAS-2B cells, showed no differences for pristine MWCNTs but significant higher cytotoxicity in BEAS-2B cells for MWCNTs-COOH at 5, 10, 20, and 40 *μ*g/mL (*P* values: 0.020, 0.020, 0.025, and 0.034, resp.).

### 3.5. Membrane Integrity (LDH Assay Results)

An increase of LDH release was found in A549 cells exposed to both MWCNTs demonstrating their capability to damage cell membrane (Figure 5(c)).

In BEAS-2B cells we found significant dose-dependent LDH release following both MWCNT exposure reaching, at the highest concentrations, values higher than those found in A549 cells (Figure 5(d)).

Mann-Whitney test, performed to compare the results of A549 in respect to BEAS-2B cells, showed for pristine MWCNTs similar cytotoxic potential at the lower concentrations and higher cytotoxicity in BEAS-2B cells at 20 and 40 *μ*g/mL with* P* values near the statistical significance (*P* = 0.050). The same test showed higher cytotoxicity for MWCNTs-COOH in A549 cells at 1, 5, and 10 *μ*g/mL (*P* values: 0.010, 0.017, and 0.034), while in BEAS-2B cells we found higher membrane damage, even if not statistically significant, at the highest concentrations.

### 3.6. Comet Assay

**Figure 6:**
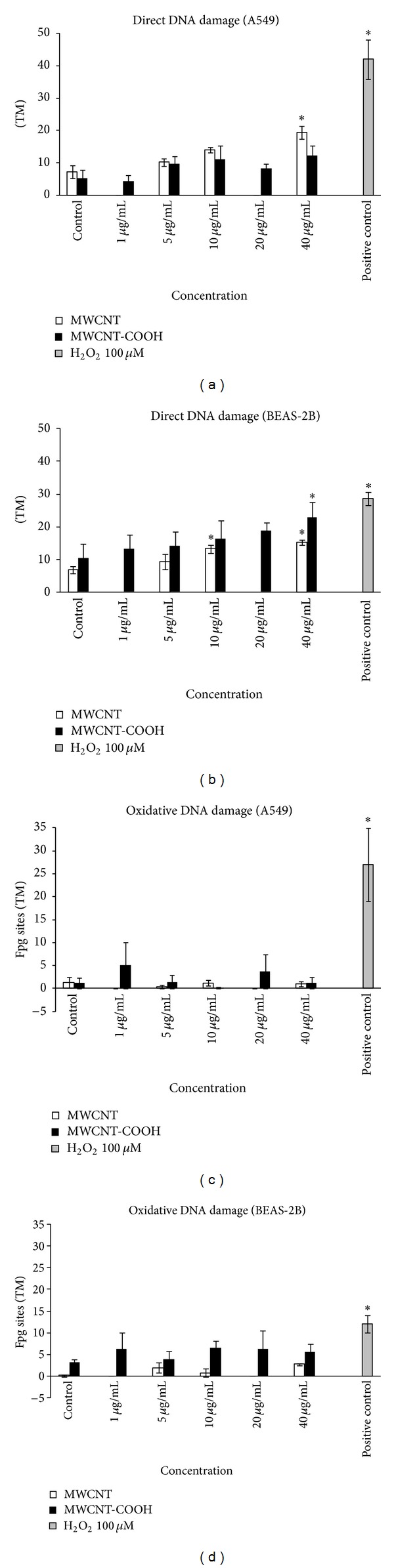
Genotoxicity of A549 ((a), (c)) and BEAS-2B ((b), (d)) cells after 24 h exposure to 5, 10, and 40 *μ*g/mL of pristine MWCNTs and to 1, 5, 10, 20, and 40 *μ*g/mL of MWCNT-COOH evaluated by Fpg-comet test. ((a), (b)) Direct DNA damage; ((c), (d)) Oxidative DNA damage. Data represent the means of three independent experiments. Hydrogen peroxide (H_2_O_2_) 100 *μ*M 30 min exposure was used as positive control. ∗*P* ≤ 0.05.

Fpg-comet test showed in A549 cells exposed to pristine MWCNTs a concentration-dependent increase of direct DNA damage in respect to control cells reaching statistical significance at 40 *μ*g/mL (*P* = 0.01) (Figure 6(a)). In the same cell line MWCNTs-COOH did not induce direct DNA damage.

BEAS-2B cells showed increase of direct DNA damage with dose-dependent trends after exposure to both the tested CNTs (Figure 6(b)).

The comparison between genotoxic effects induced by tested CNTs on the two cell lines was evaluated comparing at each concentration the TM increases of a cell line in respect to the other one. A significant difference between the two cell lines was found only for pristine MWCNTs at the highest concentration with higher TM increase value in A549 cells (12.19 versus 8.22, *P* = 0.05, Mann-Whitney).

In both cell lines a lack of oxidative DNA damage was found for pristine and functionalized MWCNTs (Figures 6(c) and 6(d)).

### 3.7. Cytokine Release

**Figure 7:**
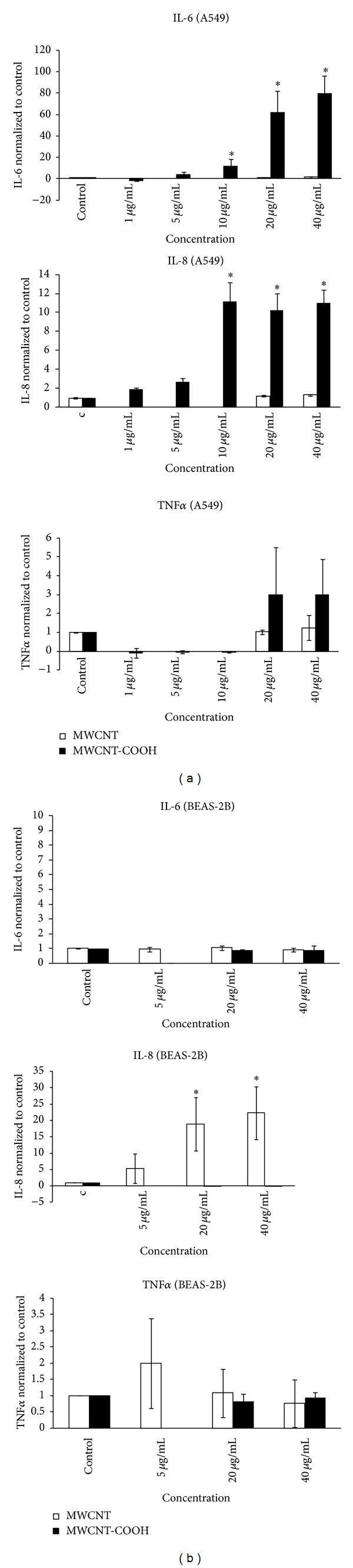
Cytokine release after 24 h exposure to 20 and 40 *μ*g/mL of pristine MWCNTs and to 1, 5, 10, 20, and 40 *μ*g/mL of MWCNT-COOH in A549 cells (a). Cytokine release after 24 h exposure to 5, 20, and 40 *μ*g/mL of pristine MWCNTs and to 20 and 40 *μ*g/mL of MWCNT-COOH in BEAS-2B cells (b). Data represent the means of three independent experiments. ∗*P* ≤ 0.05.

We found a quite different behaviour of the two kinds of cells regarding cytokine release, with A549 cells susceptible to MWCNTs-COOH and BEAS-2B cells to pristine MWCNTs. In particular, significant increases of IL-6 and IL-8 release were found in A549 cells exposed to 10, 20, and 40 *μ*g/mL of MWCNTs-COOH and no increases were detected after exposure to pristine MWCNTs (Figure 7(a)). The highest release was found for IL-6 which reached the value of 80-fold of the control at 40 *μ*g/mL of MWCNTs-COOH.

In contrast, in BEAS-2B cells we found a significant increase of IL-8 release after exposure to 20 and 40 *μ*g/mL of pristine MWCNTs and no increase of IL-6 release for both CNTs (Figure 7(b)).

Regarding TNF*α* we found in A549 cells only a slight increase of release at the highest concentrations of MWCNTs-COOH, while in BEAS-2B cells a slight increase of release was detected only at 5 *μ*g/mL of pristine MWCNTs.

## 4. Discussion

In the present study we compared the* in vitro* cytotoxic, genotoxic,and inflammatory effects of commercial pristine and COOH-functionalized MWCNTs exposing human alveolar A549 and bronchial BEAS-2B epithelial cells to low concentrations of such CNTs with the attempt to investigate their toxic effects also in relation to functionalization and the cell susceptibility. Our final aim was to identify a suitable experimental model to study CNT toxicity on respiratory system.

We found that the tested MWCNTs, furnished by the same company and synthesized in the same way, showed for the pristine form bigger dimensions, in terms of diameter and length, and consequently lower SSA.

The time-course measurements of MWCNTs adhering to the cell membrane and present inside the cells showed for BEAS-2B cells a sigmoidal curve with low uptake, within the first 4 h, and a rapid increase, starting from 8 h, with a significantly higher uptake for COOH-functionalized MWCNTs. This result is in agreement with the study of Zhang et al. [29], performed on RAW 264.7 murine macrophage cell line, that explains the enhanced cellular uptake of functionalized MWCNTs with the presence of -COOH negative surface charge that facilitates transport through the cell membrane. As suggested in the study of Zhang et al. [29], the slow uptake found in our study within the first 4 h could be explained by the fact that the MWCNTs take 2 h to deposit on the cell monolayer and then the cells started to associate with MWCNTs for up to 16 h, at which time we found saturation of cell uptake for MWCNTs-COOH differently from pristine ones that continue to enter into the cells until 24 h exposure.

The higher uptake of COOH-functionalized MWCNTs found in both cell types could explain the higher cytogenotoxic effects found for this CNT in BEAS-2B cells and the higher inflammatory response found in A549 cells. Moreover we found higher uptake from A549 cells in respect to BEAS-2B cells for both MWCNTs, with a linear time-related trend that could be due to the presence of a higher serum percentage in the specific culture medium that better disperse CNTs (particularly COOH-functionalized) facilitating the crossing of cell membrane.

The results of the present study showed on A549 cells similar low cytotoxicity for both the CNTs tested, genotoxic effects only for pristine MWCNTs, and proinflammatory response only for MWCNTs-COOH.

BEAS-2B cells were more susceptible than A549 cells to MWCNTs-COOH, in terms of cytotoxic effects (viability reduction and membrane damage induction at the highest concentrations) and in terms of genotoxicity, and to pristine MWCNTs in terms of inflammatory effects (IL-8 production). In BEAS-2B cells both the tested CNTs induced similar dose-dependent genotoxic effects even if MWCNTs-COOH showed a slightly higher genotoxic potential.

The different cytotoxic and genotoxic effects of the pristine and functionalized CNTs observed in the present study could be due, as reported by other studies, not only to the presence of -COOH group on the surface of MWCNTs that enhances cellular uptake but also to other factors such as the different dimensions in terms of length and outside diameter of CNTs that were higher in pristine MWCNTs and the tendency to agglomerate that was higher for pristine in RPMI but not in BEGM.

Several studies report the influence of the MWCNT dimensions on cytotoxicity [13, 20, 36, 37] showing different results related to the specific NM and cell type used in each study demonstrating the difficulty to identify a real association between observed effect and the physicochemical NM characteristics. In particular, Wang et al. [20] and Fenoglio et al. [36] found that thin MWCNTs were significantly more cytotoxic than the thicker ones. Also in our study, in BEAS-2B cells the thinner MWCNTs-COOH induced higher cytotoxicity in terms of viability reduction than the thicker pristine MWCNTs, although both similarly damage the membrane.

Rama Narsimha Reddy et al. [13] demonstrated, in human embryonic kidney (HEK293) cells, that viability reduction and membrane damage of four different sized MWCNTs were inversely proportional to the length and directly proportional to the surface area. Such results are in agreement with the higher cytotoxicity found in our study in BEAS-2B cells exposed to the smaller MWCNTs-COOH having higher surface area and shorter length than pristine MWCNTs. The recent study of van Berlo et al. [37] on toxicity of two types of MWCNTs with different length and entanglement/agglomeration demonstrated higher cytotoxicity and more pronounced profibrotic and inflammatory response for the longer and less agglomerated MWCNTs on murine macrophages RAW 264.7 and on mouse.

The studies evaluating the influence of functionalization on MWCNT toxicity have increased in the last years and the results are still uncertain. Bottini et al. [38] demonstrated that oxidized MWCNTs are more toxic than the more hydrophobic pristine CNTs. Also in our study on BEAS-2B cells, MWCNTs-COOH are more toxic than pristine form, showing higher cytotoxicity in respect to that found by Bottini et al. on lymphocytes, since we found a statistically significant reduction of viability after shorter exposure time and at lower concentrations. We could explain this particular behaviour with higher sensitivity of BEAS-2B cells and with the different tested MWCNTs.

Differently, Zhang et al. [29] showed that pristine MWCNTs induced higher cell viability reduction than better dispersed functionalized MWCNTs-COOH in RAW 264.7 macrophages demonstrating the influence of the agglomeration status on cytotoxicity of CNTs. Zhang et al. [29] also found that functionalized MWCNTs induce higher inflammatory response that may be associated with surface charge and agglomeration status of MWCNTs-COOH. Our results on A549 cells seem to confirm those of Zhang's study since only carboxylated CNTs induced inflammatory response that could be related to negatively charged surface, higher dispersion, and direct contact with cell membrane suggesting a functionalized MWCNT-induced inflammatory response also in lung epithelial cells. Unlike the A549 cells we found that MWCNTs-COOH did not induce any cytokine release in bronchial BEAS-2B cells, probably due to lower dispersion in the specific culture media (BEGM) and lower cellular uptake in respect to A549 cells, whereas in BEAS-2B cells we found that the pristine MWCNTs induced IL-8 release confirming previous studies on the same cell type exposed to nonfunctionalized MWCNTs [9, 11].

The images of A549 and BEAS-2B exposed cells and of the agglomerates/aggregates observed by TEM point out the higher dispersion of MWCNTs-COOH in RPMI medium with serum in respect to that of pristine ones, while in BEGM medium, containing lower amount of serum proteins and presence of growth factors and other substances, MWCNTs-COOH were less dispersed. In BEGM medium MWCNTs-COOH form different sized agglomerates, with the bigger ones that could cause high cell viability reduction damaging membrane integrity and with the smallest ones that could reach the nucleus inducing DNA damage. Membrane and DNA damages, as shown by LDH and comet results, could explain the higher potency of MWCNTs-COOH to induce reduction of viable cells in BEAS-2B together with other cytotoxic processes such as apoptosis induction that could be the result of the particular contact of such CNTs with cell membrane that triggers a cascade of reactions leading to cell death.

The higher genotoxicity of pristine MWCNTs in A549 cells found in our study could be explained by their capability to reach cell nucleus, as demonstrated by Monteiro-Riviere et al. [39] and Guo et al. [15], damaging DNA, whereas the lack of genotoxicity of functionalized MWCNTs could be explained by their inability to reach cell nucleus because, once entered into the cell, most of them remain confined in the cytoplasm in big vesicles as was found in our preliminary study by TEM analysis (data not showed) and demonstrated by Al-Jamal et al. [40] on the same cell type exposed to functionalized MWCNT-NH_3_.

The genotoxic effects found at the highest concentrations of MWCNTs-COOH on BEAS-2B cells, if related to membrane damage results, suggest that functionalized MWCNT in this cell type most seriously affect the membrane and reach more easily the nucleus than in A549 cells interacting with the DNA and damaging it after 24 h exposure.

## 5. Conclusions

The present study showed for COOH-functionalized and pristine MWCNTs different effects on the two respiratory cells used. Bronchial cells are more responsive to cytogenotoxicity of functionalized MWCNTs and to inflammatory effects of pristine, and alveolar cells are more susceptible to cytogenotoxicity of pristine and to inflammatory effects of functionalized ones.

The findings suggest the need to use simultaneously complementary end points and different cell types in* in vitro* studies on CNT toxicity. This kind of approach allowed us to evaluate the contribution of the presence of carboxylic group to the toxicity of MWCNTs on different cells of lung, which represents one of the main target organs, demonstrating a different effect of the -COOH functionalization on cytotoxicity, genotoxicity, and inflammation of alveolar and bronchial cells that show a different behaviour towards CNT insult.

The different cellular response could be related to a different interaction of the tested CNTs with the cells caused by the CNT characteristics (sizes, charge, and aggregation), also influenced by composition of the specific culture media, and by different cellular susceptibility.

The results obtained suggest then performing further studies on functionalized CNTs toxicity before starting to use them in several biomedical applications.

On the basis of our results we highlight the need to consider in* in vitro* studies on CNT toxicity, the difficulty to correlate the observed effects with the single physicochemical parameters, and the necessity to take into account other factors, such as cellular susceptibility that is influenced also by the specific cell medium, which all together contribute to the induction of the toxicity of CNTs.

## Figures and Tables

**Table 1:** Basic properties of the tested MWCNTs.

	MWCNTs	MWCNT-COOH	Testing method
Diameter (nm)	Mean 32.0 ± 15	Mean 24.5 ± 10	TEM
Length (*μ*m)	Range 0.070–7.80	Range 0.029–1.56	TEM
SSA (m^2^/g)	106.7	139.1	BET
Z potential in RPMI with 10% FBS (mV)	−9.2 ± 0.5	−10.1 ± 0.4∗	Zeta potential analyzer
Z potential in BEGM (mV)	−10.3 ± 0.6	−10.6 ± 0.9	Zeta potential analyzer
Agglomerate/aggregate size in RPMI with 10% FBS (*μ*m)	8.1 ± 4.5	4.1 ± 2.1	TEM
Agglomerate/aggregate size in BEGM (*μ*m)	5.7 ± 4.6	5.1 ± 2.9	TEM
Agglomerate/aggregate size in RPMI with 10% FBS (diameter Z average nanometers)	1542 ± 291	927 ± 231∗	DLS
Agglomerate/aggregate size in BEGM (diameter Z average nanometers)	1841 ± 617	1949 ± 700	DLS

TEM: transmission electron microscopy; BET: Brunauer-Emmett-Teller; SSA: specific surface area; DLS: dynamic light scattering. ∗*P* ≤ 0.05 MWCNT-COOH versus MWCNTs.
